# Use of laboratory animals and issues regarding the procurement of animals for research in Korea

**DOI:** 10.1186/s42826-023-00161-8

**Published:** 2023-06-01

**Authors:** Na Ahn, Jaehak Park, Sangho Roh

**Affiliations:** 1 Department of Pet Health, Kwangju Women’s University, Gwangju, 62396 Korea; 2 Laboratory Animal Medicine, College of Veterinary Medicine, Seoul National University, Seoul, 08826 Korea; 3 School of Dentistry and Dental Research Institute, Seoul National University, Seoul, 08826 Korea

**Keywords:** Animal ethics, Animal experiment, Animal procurement, Animal welfare, Laboratory animals

## Abstract

**Background:**

Laboratory animals remain critical to biomedical research, despite the increasing availability of alternative approaches. Indeed, scientists strive to reduce and refine and replace the use of laboratory animals, even in the face of public calls for ever-more stringent regulation for the protection and care of animals in research. This report outlines the current status and legal regulatory issues with regard to the procurement and use of animals for research in Korea.

**Results:**

The number of animals used for education and research purposes was increased nationwide, from 2.5 to 4.9 million in 2015 and 2021, respectively. When compared with figures from the UK, institutions in Korea were found to use more mammals such as mice and dogs. In our research, we identified three major issues concerning recent animal supply in Korea, particularly: (1) Purchase of dogs from unregistered animal supplier for a dog cloning project; (2) Purchase of dogs from an unclear source for veterinary education and training; (3) Illegal cat experiments using cats obtained from unauthorized routes.

**Conclusions:**

Our findings support the notion that alternatives to laboratory animal research should be implemented. We conclude that improvements in the regulations and guidelines for animal suppliers, together with the recent introduction of legislation will improve animal safety and wellbeing of animals in laboratory research in Korea.

## Background

Cell-based assays and computer simulation approaches using big data have increased in sophistication, such that there is evidence to show that these approaches may be feasible to replace some types of laboratory animal research work. Research with animals has nevertheless led to many critical discoveries in the field of biomedicine, for example. While controversial to the public, legal protections that govern animal research in countries around the world are in place to ensure the well-being of animals used in this research capacity. Biological research on live animals focuses on understanding health and disease; discovering biological mechanisms in health and disease, as well as developing effective drugs, vaccines, and medical devices; testing the safety of drugs, chemicals, and consumer products; conducting environmental research; and educating trainees [1]. And so, while there continues to be a move away from animal research, it remains an important element to advance human health and scientific discovery.

The government of Korea operates the Animal Protection and Welfare Division (APWD) in the Animal and Plant Quarantine Agency (APQA) under the Ministry of Agriculture, Food and Rural Affairs (MAFRA) to manage and control the use of laboratory animals and animal facility operation at the national level [2, 3]. APWD works together with the Animal Welfare Policy team of the MAFRA; it also operates the Institutional Animal Care and Use Committee (IACUC) portal website to support the IACUC operation nationwide and also offers information on the use, care, and welfare of animals to the scientists and public [4]. According to the annual report of APWD (Table 1) [4], there has been an increase in the use of animals in Korea for scientific purposes.The number of institutions operating animal facilities has also increased.

**Table 1:** National report of Korea for animal use per institution operating the animal facility

	2015	2016	2018	2019	2020	2021
No. Institutions	351	333	362	380	403	424
Total animals use (nationwide)	2,507,157	2,878,907	3,727,163	3,712,380	4,141,433	4,880,252
Animal use per institution (Average)	7142	8645	10,296	9769	10,277	11,510
Public Research institution	5884	6158	8819	6258	7217	8102
University	7322	8996	9642	10,028	9691	14,186
Medical institution	6327	7610	8648	10,352	10,619	11,064
Private company	7848	9871	11,886	11,011	11,776	11,218

Data is collated and translated from the annual reports posted in the Institute of Animal Care and Use Committee Portal [4], operated by the Animal and Plant Quarantine Agency of Korea

The data for 2017 is unavailable

For any research institute that uses laboratory animals, it is essential to ensure that the entire process is carried out by the law. This includes the animal purchase process, which must be done in a manner that is compliant with all applicable regulations. To this end, the principal investigator must confirm that the facility and staff at the institute are equipped to properly manage the animals before purchasing them for research. Indeed, prior approval by the IACUC for the purpose and quantity of laboratory animals is required [5]. In Korea, animal uses are regulated by Laboratory Animal Act (LAA) and Animal Protection Act (APA). APA was originally designed to prevent the abuse of companion animals [6]. In recent years, the policy objective and purpose have been expanded to include public health, sentiment, and confidence, through an effective system that minimizes cruelty to animals and promotes animal welfare. Moreover, the LAA aims to improve the reliability of animal research and testing through appropriate regulation and oversight of institutions, laboratory animals, and animal testing [7].

For a researcher, if their animal experiment is defined within the categories described in Article 3 of LAA [8], these may need to be obtained from registered laboratory animal suppliers, as per the following condition. “Article 3 (Object of Application): This Act shall apply to administration of animals used in testing required for any of the following subparagraphs and of animal testing facilities thereof or such: (1) development, safety control and quality control of foods, functional health foods, medical and pharmaceutical products, non-medical and pharmaceutical products, biomedicines, medical appliances, and cosmetics; (2) safety control and quality control of narcotics”. The animals for testing or research defined in Article 3 of the LAA should be supplied by one of these three routes: (1) another certified animal facility, (2) a qualified laboratory animal production facility, or (3) a registered laboratory animal supplier. In addition, laboratory animals (e.g., rodents, beagles, and primates), managed by registered laboratory animal suppliers, must be handled by these suppliers. The registered laboratory animal supplier must regularly provide information to the animal facility of the institute regarding the animal breeding group and the genetic and microbiological status of the animals as well as the clinical history (e.g., vaccination or vermicide treatment records).

One limitation of Article 3 of the LAA is that, while it clearly defines the categories of animal experimentation regulated by the LAA, many experiments are complex and are often border than those categories. However, while an institution will require researchers follow the guidelines set out by the LAA, researchers do experience difficulties obtaining animals specific to their experiments through one of those three sources. For researchers that require wild animals, farm animals, and vertebrates other than mammals, it is very difficult to source such animals from a registered laboratory animal supplier, qualified laboratory animal production facility. To overcome this, we understand that students or researchers would visit private or public farms outside of their institution for training or research, and not from the routes required by the LAA. Together with public calls for stricter regulations for animal use, researchers face great difficulty to carry out their work in compliance with fair guidelines, while also facing negative community attitudes to their work [9]. To address these issues, we have prepared a report to describe the current status of the use of laboratory animals in Korea and compares it with the status of the UK, which we recognize as using a similar number of laboratory animals for their research. In addition, we discuss animal procurement for research and education, as well as its legal regulation, by two legislative acts in Korea, the Animal Protection Act (APA) [10] and the LAA [8], and offer some suggestions for improvement.

## Results

### The use of laboratory animals in Korea

The APQA publishes online the national animal usage data and statistics for 2015, 2016, 2018, 2019, 2020 and 2021 [4]. While all information from the APQA is in Korean, we have now translated this data to the English language (Table 1). As shown, the number of institutions operating animal facilities is increasing annually, alongside an increase in the number of animals used for education and research purposes, nationwide, from 2.5 to 4.9 million in 2015 and 2021, respectively. Although the rate of increase in the use of animals per institution decreased in 2019, and increased again across 2020 and 2021.

**Fig. 1:**
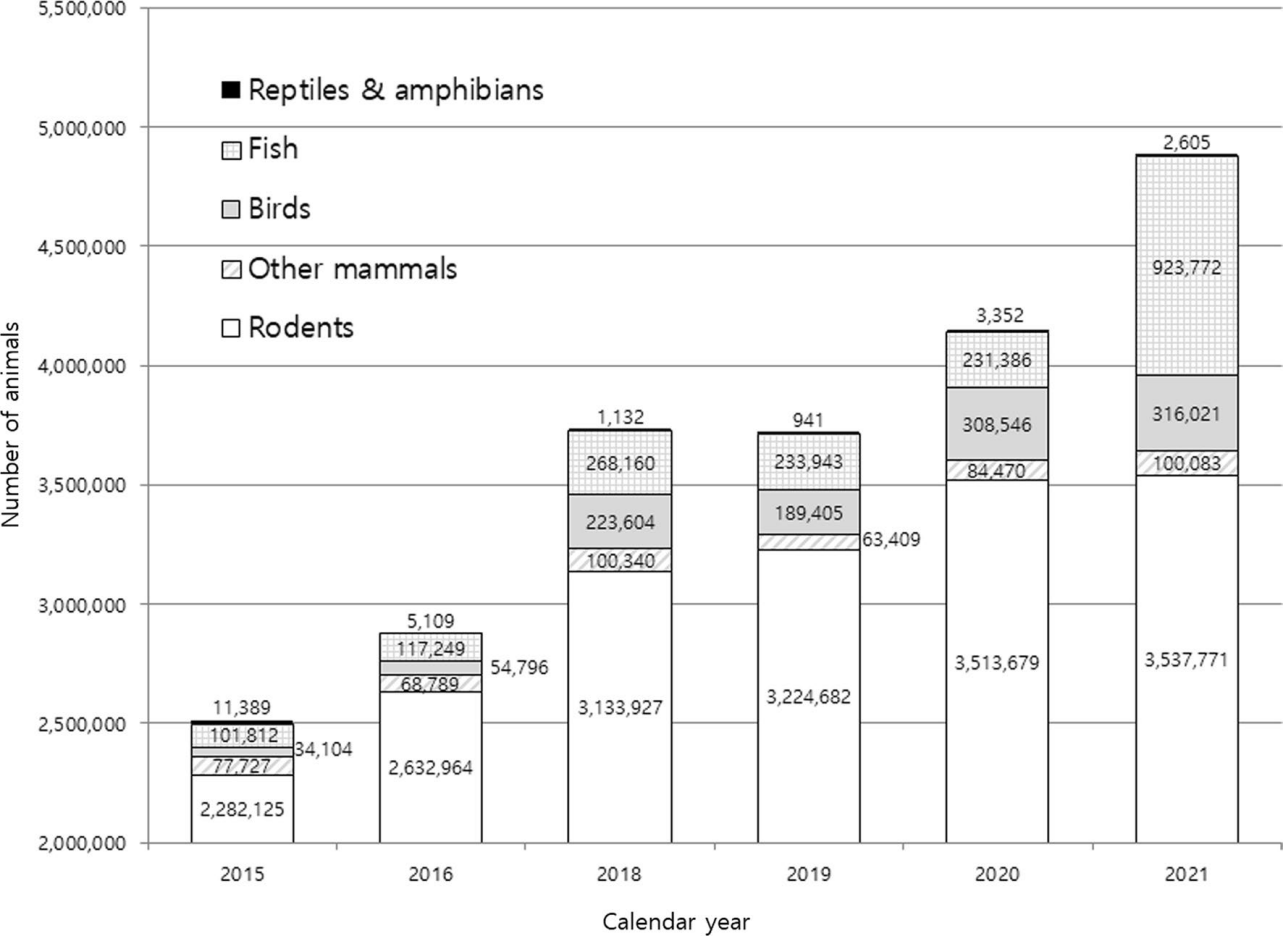
A comparison of the total numbers of animals used for research and educational purposes nationwide from 2015 to 2021. Data were collected from the national Institutional Animal Care and Use Committee portal [4]. Data for 2017 are unavailable

As expected, rodents are the most widely used animals in studies, followed by fish and birds (Fig. 1). Of the rodents, 90% were mice. Most fish and birds used were zebrafish and chicken, respectively. The use of mammals other than rodents markedly increased from 68,789 to 100,340 in 2016 and 2018, respectively. However, the number of such animals decreased to 63,409 in 2019, although rodents use increased in this period. From 2020, animal use markedly increased from the previous year. In countries such as the UK and Germany, the use of animals in research was reported to decline in 2015 and 2014, respectively [11]. According to statistics published by the UK (except Northern Ireland), animal research procedures increased after 2001, reaching a peak of 4.14 million in 2015, but it has decreased to 2.88 million in 2020. This is the lowest number of procedures carried out in a single year since 2004 [12]. In the following year, however, there was a rebound by 6% to 3.06 million in 2021 [13], which may be attributable to issues related to the pandemic and an increase in research activity during this period.

**Fig. 2:**
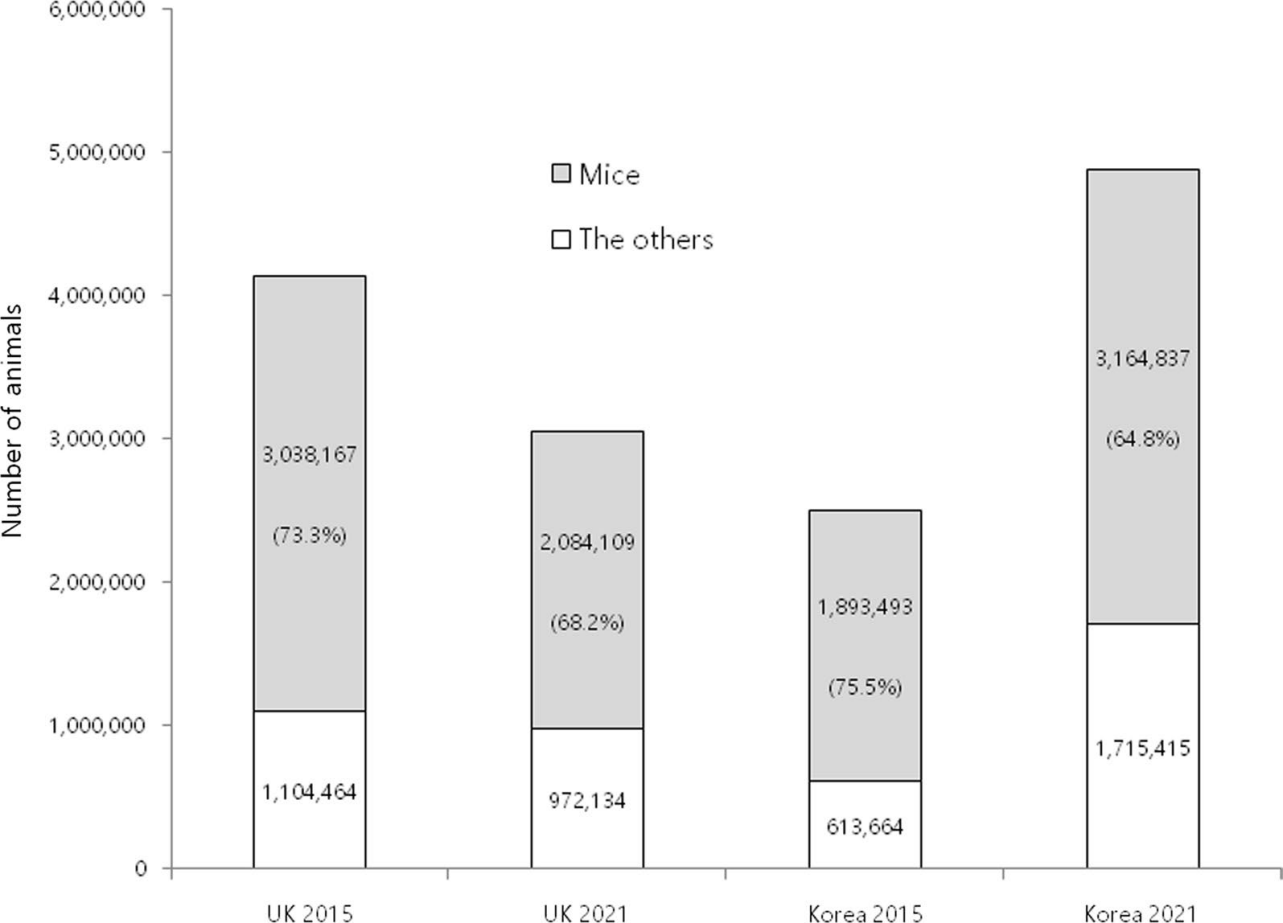
A comparison of the numbers and ratios of mice for research and educational purposes in the UK and Korea in 2015 and 2021. Data were collected from the national Institutional Animal Care and Use Committee portal [4] for Korean statistics and the Understanding Animal Research website [13] for the UK statistics

**Fig. 3:**
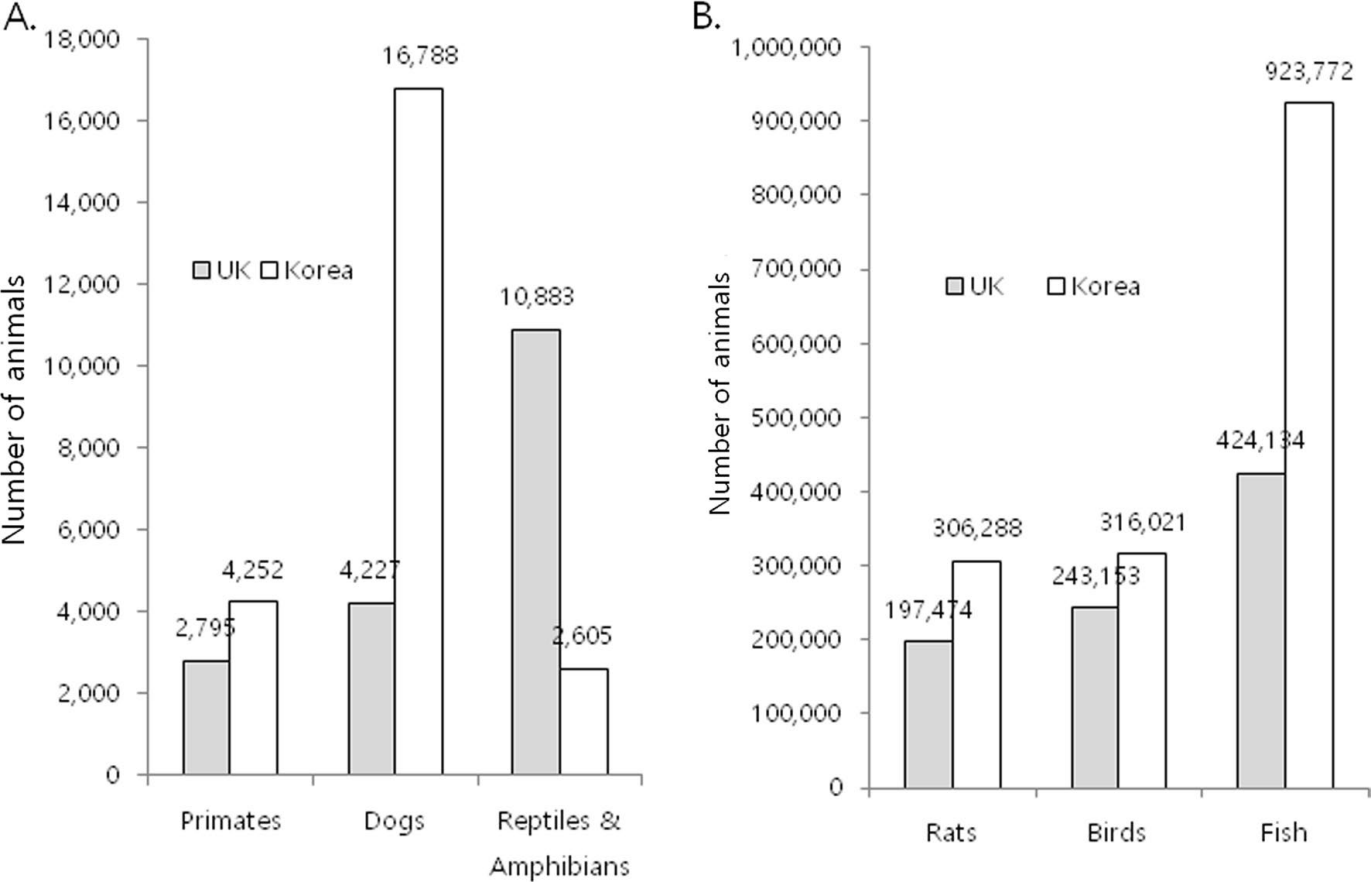
Comparisons of the numbers of various animal species for research and education purposes in the UK and Korea in 2021. **A** Primates, dogs, and reptiles/amphibians; **B** rats, birds, and fish. Data were collected from the national Institutional Animal Care and Use Committee portal [4] for Korean statistics and the Understanding Animal Research website [13] for the UK statistics

In the UK, the scientific strategy for animal experiments is changing. In fact, the Wellcome Sanger Institute, one of the world’s top genomics centers, has decided to close its 13-year-old animal research facility which supplies mice for genetic scientists worldwide [14]. The UK government also explains that, in response to the coronavirus pandemic, restrictions such as lockdown which imposed strict limits on daily life may have affected research activity at establishments as well [12]. However, no extra data were collected concerning the pandemic on its effect on the establishments. On the other hand, as shown in Fig. 1, the pandemic did not affect animal experiments in Korea. Between 2015 and 2021, while the use of laboratory animals in the UK decreased by more than 1 million, it increased in Korea during this period (Fig. 2). Research using mice and dogs was found to be more prominent across institutions in Korea, while institutions in the UK used more reptiles and amphibians (Fig. 3A, B).

**Fig. 4:**
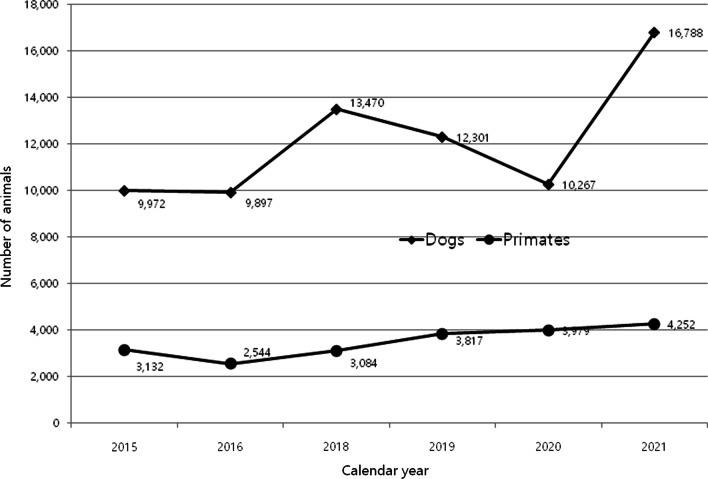
The numbers of dogs and monkeys including primates used for research and education purposes nationwide from 2015 to 2021. Data were collected from the national Institutional Animal Care and Use Committee portal [4]. Data for 2017 are unavailable

In Korea, the public is concerned about the use of companion animals and primates, and Animal Protection Organizations have always kept their eyes on the laboratory use of dogs, including beagles. According to the 2021 data we extracted, the institutions in Korea used four times more dogs for animal experiments in numbers and three times the proportion used by institutions in the UK (Fig. 3A). Primates, by the number of procedures, reached a peak of 3600 in the UK in 2015 and 2016 [13], whereas animal experiments using primates are continuously increasing in Korea by 2021 (Fig. 4). Several facilities that can manage and breed primates were recently built and are in operation in Korea, and this may contribute to the observed increase in the use of primates for research.

### Issues regarding the procurement of animals for research in Korea

Issues related to animal experiments, including illegal animal supplies, were recently discussed in Korea. Here we describe several instances that explain how the LAA is currently limited in its scope to address animal research, and outline the three major issues concerning animal supply which have recently been identified in Korea (Table 2).

**Table 2:** Recent issues related with the procurement of dogs and cats for research in Korea

Cases in point	Year and place of occurrence	Animal activist group that raised the issue
Purchase of dogs from unregistered animal supplier for a dog cloning project [16]	December, 2017 in a veterinary school’s laboratory	Korea Animal Rights Advocates (KARA)
Purchase of dogs from an unclear source for veterinary education and training [17]	March, 2018 in a veterinary school	Animal Liberation Wave (ALW)
Illegal cat experiments using cats obtained from unauthorized routes [18]	May, 2020 in a general hospital's laboratory	Beagle Rescue Network (BRN)

In 2017, Korea Animal Rights Advocates (KARA), an animal activist group, claimed that the dog cloning team, which produced world-first cloned dog [15], obtained foster and oocyte donor dogs from a dog meat farm to carry out their university-affiliated dog cloning program [16]. However, according to current legal regulations in Korea, dogs supplied to the cloning project were not included in the categories regulated by the LAA; thus, enforcement of the law was not applicable. However, activists including KARA have attempted to persuade the public and the government that all animal supplies for experiments, including dogs and cats, must be carried out by a registered laboratory animal supplier.

In another example, in 2018, a veterinary school had purchased dogs for education and training from an unclear source. The presumed source of dogs was a local dog meat shop, which could not be a registered animal supplier. Although the use of animals for veterinary education and training is out of the categories regulated by the LAA, the Animal Liberation Wave (ALW) accused a professor of this school, who is in charge of this training class, of animal cruelty and violation of the APA rules [17]. According to the ALW’s claim, the IACUC of the school failed to cross-check the address of the animal supplier, both of which was listed as the same address, a local dog meat shop.

In a third example, in 2020, a general hospital and an affiliate professor were all accused of animal abuse after allegations surfaced that they had conducted unnecessary experiments on cats and euthanized them without proper anesthesia [18]. The Beagle Rescue Network (BRN) submitted a bill of indictment and claimed that the accused parties may have used some stray or abandoned cats recovered from the streets for use in surgical experiments, violating Article 24 of the APA. In this case, animal supplies ended without prosecution, whereas matters relating to improper use of drugs on the animals currently remains on trial.

## Discussion

### Implementation of absolute or relative replacement

There is increasing momentum for researchers to replace animal experimentation with more sophisticated alternatives, including scalable, automated cell culture platforms and big-data driven approaches [19]. It is recognized that animal research in biomedical science relies heavily on resources (e.g. time, skills and experiences, animal housing, special equipment, and consumables, including food), despite its impacts in human health while minimizing the safety and well-being of animals and research practitioners [20]. In the field of toxicology, many alternatives have been proposed [21], and implementation of replacement using organoids etc. have been actively proposed even in veterinary research [22].

We found that research undertaken in Korea uses more mammals including mice and dogs when compared with the UK. It should also be noted that the use of primates is increasing in both countries. Even excluding public and activists’ calls for stricter regulation, the trend in animal use in Korea is far different from the other developed countries such as the UK and Germany [11]. These differences may encompass various factors, such as the availability of facilities that maintain specific animal species, regulations and restrictions governing animal research, and the specific research areas that necessitate the use of certain animals. For instance, the UK may have more stringent regulations on animal research than Korea, which is relevant to how some species are not used in UK research, compared to Korea. In addition, some research areas may be more prominent in Korea compared with the UK, leading to differences in the types of animals used in research. It is crucial to consider these potential factors to appreciate the use of laboratory animals between countries.

As indicated in the results, the COVID-19 pandemic resulted in a reduction in the use of laboratory animals due to challenges in operating animal facilities resulting from the UK lockdown. In the same period, in Korea, an increase in the use of animals was observed. One possibility is that the increase is attributable to the rise in demand for animal experiments as research funding for related areas, such as vaccine development, increased. Further investigation using data published by both governments will clarify this issue.

Following Russel and Burch’s concept of the 3Rs (replacement, reduction and refinement) [23], replacement alternatives are being widely discussed to avoid or replace the use of laboratory animals. This includes both absolute replacements (i.e. replacing animals with computer models or microorganisms) and relative replacements (i.e. replacing animals with lower potential for pain perception). It may be impossible to replace every animal experiment with an absolute replacement. However, there are many relative replacement options. For example, in research to understand pain in health and disease, although pain perception in fish is a matter of debate [24], the use of fish can be a part of the effort to implement relative replacement. In addition, the authority and autonomy of the IACUC must be ensured by institutions operating animal facilities to monitor the use of animals [25]. Along with researchers' efforts to apply alternative methods to animal experiments, it is essential that the government maintains productive dialogue with all relevant parties to safeguard animals in research as well as animal researchers, and promote alternatives to such experiments through improvements in legal protections and statutes that govern this human activity [26].

### Legal regulations and suggestions for revision

Purchasing animals for laboratory uses from general animal shops or suppliers, as well as sales of companion animals, currently remains beyond the scope of legal protection for animals and animal researchers. Notably, the lack of detailed health records for animals from such sources introduce confounds for research and may introduce potential infectious diseases into laboratories that use them from such sources. Yet, some animals (e.g., animals for industrial use), including chicken, are only available for purchase from registered general animal sales businesses, not from registered laboratory animal suppliers. Although most experiments or practices using industrial animals are not under the LAA regulation, some animal tests (e.g., testing of medical appliances) are included in this regulation. Purchasing industrial animals from registered animal suppliers by researchers is currently not possible, while, alongside this, specific animal breeds for research is limited by the availability of few registered suppliers. In terms of public attitudes to animal research, activists’ claims to expand the LAA regulation to most animal experiments currently under the APA regulation is of concern to researchers, and discourages them to initiate their animal research that could lead to significant biomedical breakthroughs.

We suggest that amendments to the current LAA legislature should extend only to animal studies and tests which is governed by Good Laboratory Practice, generally known as GLP. To avoid confusion and precise application of both acts, the reformation of the APA and the LAA is recommended in the future. For example, the LAA concentrates on the scientific reliability of animal experiments and the implementation of alternatives to animal testing, whereas the IACUC regulated by the APA covers all issues on animal welfare and ethics from the supply of animals to the completion of animal experiments. If the type of animal experiment falls outside of LAA regulations, the scientific issues on the experiment also apply to the APA regulations. In addition, rather than regulating laboratory animal suppliers through legal means, enhancing education on ethical animal handling and treatment offers a more sustainable approach for researchers, and for the public to benefit from biomedical research discoveries using this approach that improve our health and well-being.

## Conclusions

We find that institutions in Korea use comparable numbers of laboratory animals, when compared with data from the UK, yet more mammals such as mice and dogs are used in Korean research. Alternatives should be implemented to reduce or replace the use of laboratory animals. We suggest reforms in the regulations and guidelines on animal supplies, consistent with the introduction of recent legal issues regarding the procurement of animals that will improve the proper use of laboratory animals.

## Methods

National data for the use of animals from 2015 to 2021 was collected from the annual press release posted on the IACUC portal, which is open to the public and free to access. APQA did not post the 2017 press release online; thus, this present study could not present data for 2017. The UK statistics for the use of animals in 2015 and 2021 posted by the Understanding Animal Research (UAR) [13] were compared with the Korean statistics of the same years. UAR permitted the use of materials from its website. The UK government prefers to use the term “the number of procedures,” not “the number of animals,” because some animals may be used more than once, i.e., “re-used” in certain circumstances. Therefore, the number of procedures is usually slightly higher than the number of animals used. However, statistics from the Korean source we used cannot distinguish this, and the statistics posted in UAR were directly compared with Korean statistics.

All national legal information regarding to the use of animals and supplies was obtained from the Korean Law Translation Center website, which is operated by the Korea Legislation Research Institute (KLRI) [27]. To obtain the interests and views of animal activists and the public about the issues regarding the use and supply of companion animals for research, the articles and editorials produced by various media were collected online and recent three cases were selected for discussion among the collected information.

## Data Availability

Data of the study may be available upon reasonable request to the corresponding authors.

## Abbreviations

ALW: Animal Liberation Wave
APA: Animal Protection Act
APQA: Animal and Plant Quarantine Agency
APWD: Animal Protection and Welfare Division
BRN: Beagle Rescue Network
GLP: Good Laboratory Practice
IACUC: Institutional Animal Care and Use Committee
KARA: Korea Animal Rights Advocates
KLRI: Korea Legislation Research Institute
LAA: Laboratory Animal Act
MAFRA: Ministry of Agriculture, Food and Rural Affairs
UAR: Understanding Animal Research
